# Generate-Boost: study protocol for a prospective, multicenter, randomized controlled, double-blinded phase II trial to evaluate efficacy and safety of bortezomib in patients with severe autoimmune encephalitis

**DOI:** 10.1186/s13063-020-04516-7

**Published:** 2020-07-08

**Authors:** Jonathan Wickel, Ha-Yeun Chung, Stephanie Platzer, Thomas Lehmann, Harald Prüss, Frank Leypoldt, Albrecht Günther, André Scherag, Christian Geis, on behalf of the GENERATE Study Group

**Affiliations:** 1 Section of Translational Neuroimmunology, Hans Berger Department of Neurology, Jena University Hospital, Am Klinikum 1, 07747 Jena, Germany; 2 Center of Clinical Studies, Jena University Hospital, Jena, 07747 Germany; 3 German Center for Neurodegenerative Diseases (DZNE) Berlin and Department of Neurology and Experimental Neurology, Charité – Universitätsmedizin Berlin, 10117 Berlin, Germany; 4 Neuroimmunology, Institute of Clinical chemistry and Department of Neurology, University Hospital Schleswig-Holstein and Christian-Albrechts-University, Kiel, 24105 Kiel, Germany; 5 Institute of Medical Statistics, Computer and Data Sciences, Jena University Hospital, Jena, 07743 Germany

**Keywords:** Autoimmune CNS disorder, Autoimmune encephalitis, Bortezomib, Autoantibody, Plasma cell, NMDA receptor, LGI1, CASPR2, Rituximab, Disability

## Abstract

**Background:**

Autoimmune encephalitis is a new spectrum of autoimmune disorders of the central nervous system (CNS), which are characterized by pathogenic autoantibodies against neuronal surface antigens. Clinical presentations range from acute to subacute encephalopathy with neurological and psychiatric symptoms, and life-threatening autonomic dysfunction in severe cases. There exist no approved therapies nor is data available from controlled clinical trials. Patients are usually treated with diverse combinations of immunotherapy. However, effect of immunotherapy on antibody-producing cells and thus on levels of pathogenic autoantibodies is insufficient. Therefore, therapeutic response is sometimes prolonged with necessity of long-time intensive care treatment and also irreversible deficits occur in severe cases. This trial will investigate the efficacy and safety of bortezomib, a proteasome inhibitor known to selectively deplete plasma cells, in patients with severe autoimmune encephalitis who have been treated with rituximab with insufficient response.

**Methods:**

Generate-Boost is an investigator-initiated, multicenter, double-blinded, randomized controlled phase II trial which will be conducted in specialized neurological hospitals within the GENERATE (GErman NEtwork for Research on AuToimmune Encephalitis) network in Germany. Adult patients with severe autoimmune encephalitis (modified Rankin scale, mRS ≥ 3), autoantibodies against neuronal surface antigens, and pretreatment with rituximab are eligible for study participation. Fifty patients will be randomized 1:1 and undergo up to 3 cycles (each 21 days with 4 s. c. applications) of bortezomib or placebo. All patients will receive concomitant medication with dexamethasone, acyclovir and co-trimoxazole. The primary efficacy endpoint is the mRS score 17 weeks after first treatment application. Secondary endpoints are neurocognitive function, antibody titers, markers of neuronal cell damage, length of ICU/hospital stay, and mRS and Glasgow coma scale scores throughout the trial up to week 17. General and bortezomib-specific adverse events are monitored continuously.

**Discussion:**

The expected outcome of the study is to obtain first reliable data on a hypothesis-driven therapeutic option in severe and difficult-to-treat autoimmune encephalitis. If treatment with bortezomib is beneficial in these cases, this will be the basis for implementation in the current guidelines.

**Trial registration:**

Clinicaltrials.gov, NCT03993262. Registered June 20, 2019;

German Clinical Trials Register, DRKS00017497.

## Background

Autoimmune encephalitis denotes a recently defined [1] and increasing group of rare, inflammatory disorders of the CNS with an incidence of up to 1/100.000 person-years [2]. Autoimmune encephalitis is characterized by highly specific autoantibodies targeting membrane surface proteins of neurons. So far, more than 15 target antigens have been described which are mostly expressed in synaptic locations [3]. Encephalitis mediated by antibodies against the *N*-methyl-d-aspartate (NMDA) receptor [4, 5] is the most frequent subtype followed by encephalitis with antibodies to the synaptic linker protein leucine-rich, glioma inactivated 1 (LGI1) (5). Although clinical presentations and associated symptoms are different between autoantibody-defined subtypes, patients with autoimmune encephalitis develop acute to subacute encephalopathy with memory dysfunction, seizures, behavioral changes, psychotic episodes, sometimes movement disorders and especially in severe case therapy-refractory status epilepticus and life-threatening autonomic dysfunction [6]. No approved therapies exist and patients are empirically treated with combinations of immunotherapies based on retrospective and cohort studies or case reports [1, 7]: Plasma exchange or immunoadsorption together with i.v. (and sometimes oral) steroids (so-called “first-line therapy”) and-in severe cases-with B cell depletion or cyclophosphamide (so-called “second-line therapy”), respectively [5]. Although treatment response is favorable and timely in up to 50% of cases, a significant proportion of up to 25% of patients have severely prolonged treatment response or are refractory to treatment [7]. Many of these patients require long-term in-hospital stay, mostly on the intensive care unit (ICU) due to life-threatening complications, e.g., hypoventilation or autonomic disturbances [8]. It is hypothesized that long-living systemic but also intrathecal, meningeal, and parenchymal plasma cells surviving in plasma-cell niches are responsible for pathogenic antibody production in refractory cases [3, 9]. The B cell depleting therapeutic antibody rituximab targets CD20-positive B cells and may therefore reduce the number of short-lived plasma cells that originate from activated B cells. However, the CD138-positive plasma cells lack the CD20 surface receptor, are not targeted by rituximab, and are resistant to other applied immunosuppressive treatments [10, 11].

Bortezomib is a proteasome inhibitor and interferes with the NF-κB and the ubiquitin-proteasome pathway [12]. By inhibiting the proteasome function, it preferentially affects cells with high-protein synthesis, such as plasma cells, leading to apoptotic cell death. It has been shown that proteasome inhibition by bortezomib induces depletion of long-lived plasma cells in systemic autoimmune disorders, e.g., systemic lupus erythematosus resulting in reduction of pathogenic autoantibody levels and in clinical improvement [13]. In a first retrospective case series, the therapeutic potential of bortezomib was evaluated in 5 patients with severe and therapy-refractory NMDA receptor encephalitis [10]. Bortezomib induced clinical improvement and disease remission together with reduction of titers of antibodies to the NMDA receptor. Although bortezomib is generally believed not to cross the blood brain barrier in the normal state, it is conceivable that during chronic brain inflammation and impaired blood-brain barrier also intrathecal and parenchymal plasma cells in the CNS are accessible to proteasome inhibition [10].

We performed a standardized database search using the US National Library of Medicine to identify published and ongoing trials investigating treatment options in autoimmune encephalitis. By using the search terms “autoimmune encephalitis” or “NMDA receptor encephalitis” and “clinical study” or “clinical trial,” we did not identify published results from controlled clinical trials. We found the description of a study protocol of a multicenter randomized controlled trial regarding the role of intravenous immunoglobulin (IVIG) in the treatment of children with encephalitis (IgNiTE; NCT02308982). This study investigates IVIG in encephalitis from any cause including encephalitis of autoimmune but also of infectious etiology. We also found a study description of a phase II double blinded placebo-controlled trial to investigate the effect of IVIG followed by rituximab vs. placebo in antibody-associated psychosis (first presentation or relapse; NCT03194815). Furthermore, we identified a randomized, placebo-controlled study investigating the efficacy of the B cell depleting agent ocrelizumab in autoimmune encephalitis (NCT03835728). Additionally, several open label trials exist to investigate potential benefit of erythropoietin (NCT03004209), IVIG (NCT04175522), low-dose IL-2 (NCT02714959), and early plasma exchange (NCT 03542279) in autoimmune encephalitis. Another open-label interventional trial analyzes the role of vigabatrin on seizure control in refractory autoimmune encephalitis (NCT03003143). In a prospective observational case control pilot study, the effect of immunoadsorption or plasma exchange has been investigated in the treatment of autoimmune encephalitis [14]. Additional search for “bortezomib” in combination with the clinical term autoimmune or NMDA receptor encephalitis in MEDLINE gave two results of retrospective case reports [10, 15]. None of these studies overlaps with the planned intervention in Generate-Boost.

### Research question

Motivated by the biological rationale of plasma cell depletion using bortezomib, we hypothesize that up to three cycles of subcutaneous (s.c.) treatment with bortezomib as compared to placebo will lead to an improvement of severe disease symptoms in patients with antibody-positive autoimmune encephalitis that have insufficiently responded to standard treatment including rituximab.

## Methods/design

### Overview of trial design

Generate-Boost is an investigator-initiated, multicenter, double-blinded, randomized controlled phase II trial (EudraCT: 2019–001423-12; see also supplementary Table 1 for detailed description of the WHO data set). The study is funded by the German Federal Ministry of Education and Research (BMBF, 01GM1908E). Patients with autoantibody-positive severe autoimmune encephalitis will be randomly allocated to receive up to three 21-day cycles (each cycle with 4 s.c. applications) of bortezomib (experimental therapy arm) or 0.9% sodium chloride (saline, NaCl, placebo control group). Approval has been obtained from the leading Institutional Review Board at Jena University Hospital (ref. 2019-1523-AMG_ff), the relevant Institutional Review Boards of the participating study sites, and the Federal Institute for Drugs and Medical Devices (ref. 4043753). We provide a “Standard Protocol Items: Recommendations for Interventional Trials” (SPIRIT) checklist as Additional file 1 [16].

Generate-Boost will be conducted at several neurological centers of the GENERATE network (German Network for Research on Autoimmune Encephalitis; www.generate.net, see Table 1). Fifty patients are planned to be included. A flowchart of the planned enrolment, interventions, and assessments is shown in Fig. 1.

**Table 1:** List of participating study centers

#	Site/address
01	Jena University Hospital, Department of Neurology Am Klinikum 1, 07747 Jena, Germany
02	University Hospital Schleswig-Holstein Campus Kiel, Neuroimmunology, Department of Neurology and Inst. of Clinical Chemistry Arnold-Heller-Str. 3, Haus 41, 24105 Kiel, Germany
03	University Hospital Ulm, Department of Neurology Oberer Eselsberg 45, 89081 Ulm, Germany
04	Charité – University Medicine Berlin, Department of Neurology Charitéplatz 1, 10117 Berlin, Germany
05	University Hospital Münster, Department of Neurology Albert-Schweitzer-Campus 1, 48149 Münster, Germany
06	University Hospital Würzburg, Department of Neurology Josef-Schneider-Straße 11, 97080 Würzburg, Germany
07	Medizinische Hochschule Hannover, Department of Neurology Carl-Beuberg-Str. 1, 30625 Hannover, Germany
08	Ludwig-Maximilians-University Munich, Institute of Clinical Neuroimmunology and Department of Neurology Marchioninistraße 15, 81377 München, Germany
09	Ruhr-University Bochum, St. Josef Hospital, Department of Neurology Gudrunstr. 56, 44791 Bochum, Germany

**Fig. 1:**
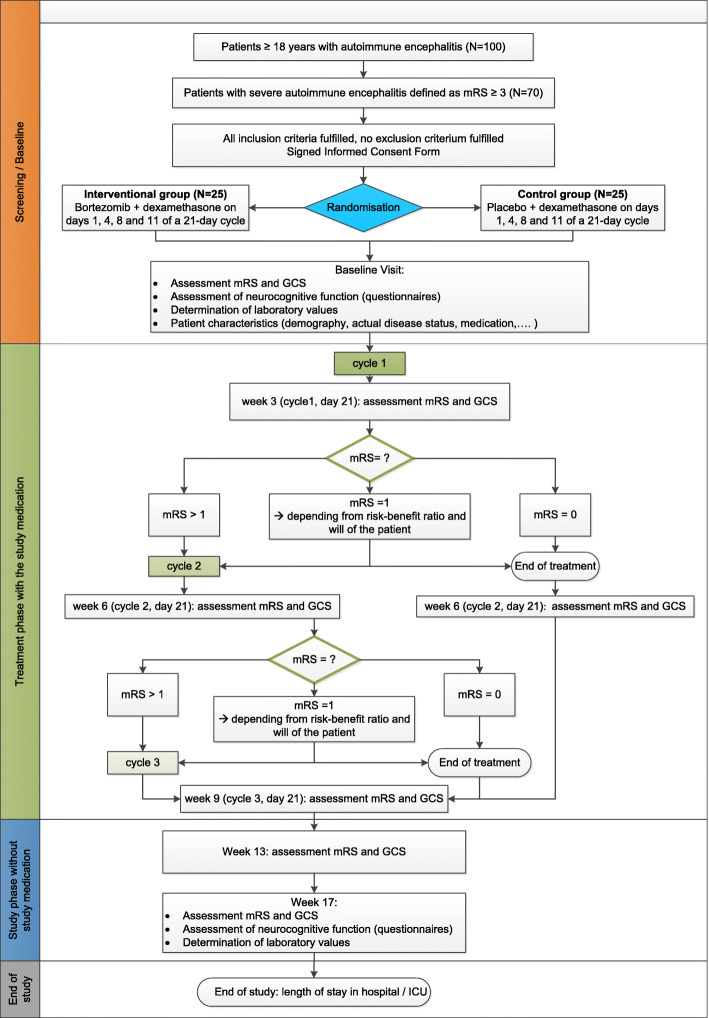
Flowchart of planned enrolment, interventions and assessments

### Aims

#### Primary objective

The primary objective is to determine if treatment with bortezomib will lead to a more effective and faster therapeutic response as compared to standard immunotherapy as measured by improvement on the modified Ranking scale (mRS). The mRS is a well-established scale which measures dependence in daily activities and the degree of disability. Originally used primarily in stroke patients [17], it has been established in other neurological diseases as well, including autoimmune encephalitis [18]. A reduction of the mRS score indicates an increase of independence and autonomy. Only patients with an mRS score of ≥ 3 will be included in GENERATE-Boost. mRS score of 3 indicates moderate disability in which the patient requires help in activities of daily living, but is able to walk unassisted [17]. The primary endpoint of Generate-Boost is the impact of bortezomib on the mRS score 17 weeks after first application of study medication.

#### Secondary objectives

Secondary objectives aim at analyzing the influence of bortezomib on additional disease-relevant clinical parameters, e.g., neurocognitive function, on disease severity at further time points during the study period, and on paraclinical surrogate parameters, e.g., laboratory findings of auto-antibody and vaccine titers/intrathecal synthesis, analysis of changes in central and systemic clonality of immune cells, and markers of neuronal damage (for detailed overview on endpoints definition see also Supplementary Table 2).

In detail, the secondary endpoints will evaluate the influence of bortezomib on:

- mRS scores 3, 6, 9, and 13 weeks after the first application of study medication
- Glasgow coma scale score 3, 6, 9, 13, and 17 weeks after first application of study medication
- Duration of hospital/ duration of ICU stay
- Neurocognitive function (Montreal Cognitive Assessment Test, MoCA; Mini Mental Status Examination, MMSE; Neuro Psychiatric Inventory, NPI; Rey Auditory Verbal Learning Test, RAVLT) at baseline and 17 weeks after first application of study medication
- Absolute and relative change in auto-antibody and vaccine titers in serum and in the cerebrospinal fluid as well as intrathecal synthesis (CSF; at baseline and 17 weeks after the first application of study medication)
- Immunostatus (differential blood count, IgG, IgM, and IgA fractions)
- Markers of neuronal damage (neurofilament light chain, NFL; Glial fibrillary acidic protein, GFAP; TAU; ubiquitin carboxy-terminal hydrolase L1; UCH-L1) in the serum at baseline and 17 weeks after the first application of study medication
- Change in clonality analysis of B and T cell receptors from bulk NGS sequencing from CSF cells and PBMCs.

Furthermore, we assess the following safety endpoints:

- (Serious) adverse events within 17 weeks after the first application of study medication.
- To distinct bortezomib safety: polyneuropathy, increase of liver enzymes, hemotoxicity, gastrointestinal toxicity, infectious events

### Eligibility criteria

Patient inclusion criteria are:

- Age 18 years or older
- Clinically diagnosed severe autoimmune encephalitis (mRS ≥ 3)
- Antineuronal surface autoantibodies (e.g., NMDA receptor, LGI1, CASPR2, others) in CSF or serum (determined within maximum 4 weeks before randomization)
- Pretreatment with rituximab
- Written informed consent of the patient or legal representative
- Negative pregnancy test in women of child-bearing potential (until 2 years after menopause)

The exclusion criteria are:

- Pregnancy/lactation
- Acute infiltrating pulmonary disease
- Acute infiltrating pericardial disease
- Malignant tumor with ongoing chemotherapy
- Concomitant participation in other interventional study
- Preceding participation in Generate-Boost
- Known hypersensitivity to bortezomib or dexamethasone
- Ongoing other immunotherapy except for that implicated in the study protocol

### Randomization and blinding

Patients will be centrally randomized in a 1:1 manner. The randomization list will be generated by the Center for Clinical Studies (ZKS) of the Jena University Hospital using nQuery 7.0 in advance to ensure allocation concealment. To achieve balanced distributions for pretreatment factors, we apply stratified block randomization of variable block length (stratum: center). For each study center, a separate randomization list will be allocated to the respective hospital pharmacy which is responsible for the blinded treatment allocation according to the randomization list. The study personnel at the site (physicians, study nurses, study coordinators) are blinded. The local pharmacy provides the ready-to-use syringe with placebo or verum with an appropriate label giving the study name and ID. Placebo will be provided in the same volume as the verum would have according to the required dosing. The local pharmacy is instructed to maintain any information on the identity of the trial drug strictly confidential. The site is provided a sealed randomization list for emergency unblinding, if the identity of the trial drug is absolutely necessary for the further treatment of the patient. The monitor will check this list for any manipulations at every on-site visit. After completion of the trial, this list will be collected by the monitor, so that even after the trial no unblinding can occur.

Each randomized patient will be part of the intention-to-treat (ITT) analysis set. Sub-group analyses will be performed according to the type of anti-neuronal antibodies. Patients will be excluded from the “per-protocol” analysis population when one or more of the following conditions occur during the study:

- Patient did not receive study medication
- Patient received plasma exchange, immunoadsorption, or any other immunotherapy other than those designated in the study protocol (bortezomib and dexamethasone)
- Patients who developed severe adverse reaction during the first application of study medication and who had to discontinue study medication for that reason
- Patients who received an additional application of study medication despite treatment with study medication was regularly terminated
- Patients that were set on chemotherapy during the trial

### Interventions

**Fig. 2:**
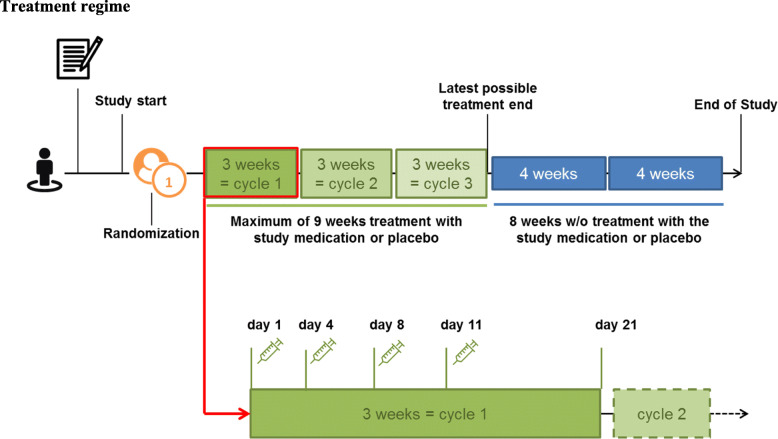
Treatment regime

Patients will be randomly allocated to receive bortezomib (experimental therapy group) or sodium chloride (saline, NaCl 0.9%, placebo control group). The study will investigate the effects of up to 3 cycles of bortezomib consisting of 4 s.c. applications of bortezomib (1.3 mg/m^2^ body weight on day 1, 4, 8, and 11 of a 21-day cycle starting at weeks 0, 3, and 6) versus respective NaCl 0.9% treatment (Fig. 2). Laboratory tests and special awareness for neuropathy are required before every administration of the study medication as dose adaptions may be needed. For details on dose adaption, see Table 2.

**Table 2:** Dose adaption of study medication

Neuropathy	Grade 1 with pain or Grade 2 (moderate symptoms; limiting instrumental activities of daily living, ADL)	Reduction of dose level to 1.0 mg/m^2^ BSA
	Grade 2 with pain or Grade 3 (severe symptoms; limiting self-care ADL)	Suspension of treatment; if resolved, continuation with reduced dose (one dose level below prior treatment dose); if suspension persists for > 2 weeks, study treatment has to be discontinued.
	Grade 4 (life-threatening consequences; urgent intervention indicated)	Discontinuation of study treatment
Bilirubin	> 1.5× upper limit of normal (ULN)	Reduction of dose level to 0.7 mg/m^2^ BSA; if additional treatment cycle: depending on tolerability increase of dose level to 1.0 mg/m^2^ BSA or further reduction to 0.5 mg/m^2^ BSA
Thrombopenia/neutropenia	Thrombopenia with < 25.000/μl	Suspension of treatment until resolved If suspension persists for > 2 weeks, study treatment has to be discontinued.
	Neutropenia with fever	
	Neutropenia with < 750/μl	

**Fig. 3:**
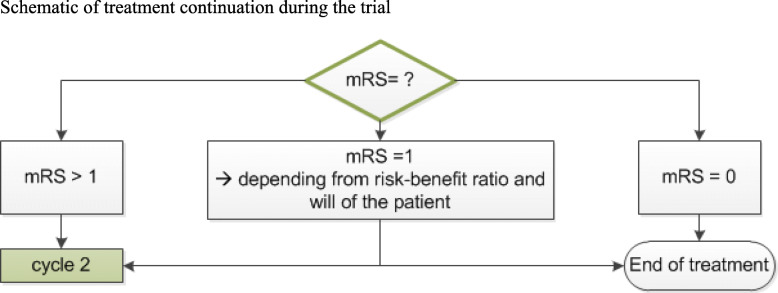
Schematic of treatment continuation during the trial

At the days of the study medication injections, additional 20 mg dexamethasone will be administered orally. Patients will also receive antiviral and antibiotic prophylactic therapy until 3 weeks after the last treatment with the study medication: acyclovir (2 × 400 mg daily) and co-trimoxazole (960 mg 2x per week). If no oral intake is possible, treatment will be administered i.v. Additional immunosuppressive or immunomodulatory treatment to study medication is prohibited. As bortezomib is metabolized via CYP3A4, drugs which are metabolized by the same enzyme should not be used in study patients. These drugs include, e.g., carbamazepine, phenytoine, phenobarbital, rifampicin, and St John’s wort. If patients show a profound clinical relief after the first or second cycle of the study medication (mRS = 1), further treatment with study medication can be suspended on decision of the blinded treating physician. If mRS = 0, no further treatment with study medication will be applied (Fig. 3). These patients will not be excluded from the analysis. For more details, see the schedule of enrolment, interventions, and assessment (Table 3).

**Table 3:**
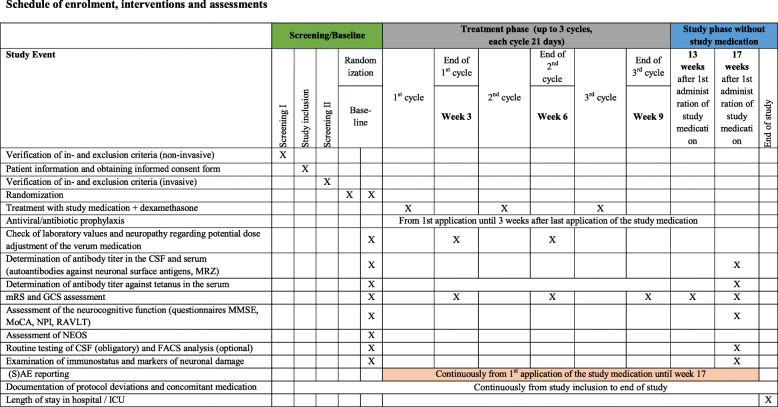
Schedule of enrolment, interventions, and assessments

### Clinical scales and assessment of disease severity

Disease severity will be rated using the mRS as well as the Glasgow coma scale (GCS) and will be performed by the study team in the centers before randomization, after each cycle of study medication on weeks 3, 6, and 9 and in the follow-up on weeks 13 and 17. Neurocognitive performance will be assessed at baseline and week 17 using the Mini-Mental State Examination (MMSE), the Montreal Cognitive Assessment (MoCA), the Neuropsychiatric Inventory (NPI), and the German equivalent of the Rey Auditory Verbal Learning Test (RAVLT). The anti-NMDAR Encephalitis One-Year Functional Status (NEOS) score will be assessed at baseline (independent from the subtype of detected antineuronal antibodies). Antineuronal antibody titers and additional markers in serum and CSF will be determined at baseline and week 17 after the start of treatment. For more details, see the schedule of enrolment, interventions, and assessment (Table 3). Additional routine blood laboratory parameters will be assessed before each application of study medication, at day 21 after each cycle, as well as at week 17 to exclude drug-induced side effects, e.g., thrombopenia and leukopenia as well as elevation of liver enzymes. Furthermore, the length of the ICU stay after start of the study medication will be evaluated at the end of the study. (Serious) adverse events will be monitored throughout the whole study period starting with the first application of the study medication up to week 17.

### Laboratory parameters and analysis

The following parameters will be analyzed at baseline and week 17:

#### Antineuronal antibody titers

Detection of antineuronal autoantibodies (e.g., to the NMDA receptor, AMPA receptor, GABA-B receptor, LGI1, and others) and titer measurement is performed at the study centers using routine CSF and serum tests. To ensure comparability within the study centers, additional measurement will be done in the central laboratory of the University Hospital Schleswig-Holstein, Institute of Clinical Chemistry. Validated immunohistological procedures [19], commercially available, antigen-specific test arrays (e.g., Euroimmun Mosaic) will be used for analysis. Additionally, fixed and life-in-house cell-based assays with transfected human HEK293T cells will be used for confirmation of commercial assays and if antigen-specific test arrays are not available for a specific antibody.

#### MRZ reaction and tetanus titer

In the central laboratory, CSF and serum antibody titers to measles (M), rubella (R), and varicella zoster (Z) will be determined in CSF and serum and intrathecal synthesis calculated. Tetanus antibody titers will be measured in serum only.

#### Additional biomarkers

In the central laboratory, Single Molecule Array (Simoa®) measurements of neurofilament light chain (NFL), glial fibrillary acidic protein (GFAP), ubiquitin carboxy-terminal hydrolase L1 (UCH-L1), and Tau will be performed in serum from all available time points.

#### Peripheral blood mononuclear cells and CSF cells

In the study sites, CSF cells and peripheral blood mononuclear cells (PBMCs) will be harvested. Frozen PBMCs and CSF cells will be sent to the central laboratory for further analysis, i.e., bulk RNA next-generation sequencing. Cells will be acquired at baseline and at week 17 to analyze B and T cell receptor repertoire and analysis of clonality before and after treatment.

### Data management

Trained staff at each study site will conduct data entry in electronic case report forms (eCRFs). Data are collected on the servers of the ZKS at Jena University Hospital via web-based data capture software (OpenClinica, LLC, Waltham, MA USA). “OpenClinica®” meets all regulatory requirements (GCP, 21CFRPart11). It has an integrated audit trail that records any kind of data changes automatically. In order to ensure a pseudonymized analysis of data, each patient data set is given a unique patient identification number when being entered into the study data base. Only the specific study site, monitors and authorized third parties (auditors, inspectors) have access to personalized patient data.

### Patient safety monitoring

An independent Data and Safety Monitoring Board (DSMB) is installed. It comprises an independent immunologist, an independent statistician, and an independent medical ethicist. All members are independent from the sponsor and claim no competing interests. The DSMB will monitor and supervise the progress of the trial at regular intervals (interim safety analyses) and evaluate the safety data. Therefore, the DSMB receives information about the trial progress, amendments, and listings of safety-relevant items including serious adverse events (SAEs) and suspected unexpected serious adverse reactions (SUSARs) continuously during the study. Interim safety analyses are planned along with the legally required annual safety report to inform the DSMB. The DSMB will give recommendations to the sponsor and coordinating investigator whether to continue or to stop the trial or to modify the trial protocol. AEs will be collected systematically, regardless of the expectedness, frequency, and severity. AEs will be recorded in the CRF and reported throughout the trial. SAE reporting and management will follow the Standard Operation Procedures (SOPs) at the ZKS Jena. SAEs have to be reported immediately (within 24 h the latest) to the pharmacovigilance section of the ZKS Jena. AEs and SAEs will be assessed for expectedness compared to the professional information of the trial drug bortezomib. Unexpected AEs will be listed in the next update of the Investigator’s Brochure. Unexpected SAEs which fulfill the criteria for a SUSAR will be reported to the ethics committee and the federal authority within the legal deadlines. As this trial is the first in which the effect of bortezomib in patients with autoimmune encephalitis is evaluated, all AEs will be reported in the trial publication.

### Quality assurance and safety

Site selection was based on a registry of patients with autoimmune encephalitis of the GENERATE network assuring eligibility of the participating center concerning feasibility of recruiting and standards of patient care. Quality of patient care and handling will be approved by checking for DIN EN ISO 9001 or equivalent certification procedures and certificates. Results will be formally reported and in doubt of eligibility, the center might be withdrawn from study participation.

All trial-related processes including monitoring will follow the SOPs of the ZKS at the Jena University Hospital. The first regular monitoring visit will take place shortly after the first treatment cycle of the first patient in every single center. Following the first visit, centers will be visited approximately three times. The frequency is depending on the recruitment rate of the respective center. The monitoring reports will be reviewed by the project manager and the Coordinating Investigator/Sponsor Representative and appropriate measures will be taken, if necessary. The information recorded in the eCRFs at the trial site is regularly and systematically checked for completeness, consistency and plausibility by routines implemented in data capture software and by central monitoring. Agreement of study data with source data and compliance with the informed consent process are verified by external monitors. Safety of the study medication is assessed by reporting of adverse events, SAEs, and SUSARs. Particular attention will be paid on possible bortezomib-induced side effects, e.g., thrombopenia, liver toxicity, neuropathy, and increased susceptibility for infection. According to German regulations, safety reports are forwarded to the authorities and ethics boards. The Sponsor Core Unit (CUS) of the Friedrich-Schiller-University will conduct an internal audit at the site Jena after inclusion of the first patient at this site. On request of the Coordinating Investigator/Sponsor Representative, the CUS will conduct audits also at external study sites. Additionally, the lower federal authorities in Germany (Landesbehörden) may inspect the respective trial sites for appropriate trial conduct and adherence to GCP and the respective German laws. The DSMB will receive a descriptive analysis regularly to assess the safety of the study intervention. Protocol modifications and amendments will be communicated to the investigators and to the DSMB by the ZKS at the Jena University Hospital. Patients will be informed by the respective centers using updated informed consent forms.

### Sample size/power calculations

Autoimmune encephalitis is a group of rare diseases and there is no data available from RCTs for this patient population. Several special recommendations for clinical studies in rare diseases have been provided which include considerations on how to adapt standard sample size planning [20]. We decided to follow the idea of describing the information provided for a given sample size of 2 × 20 patients. First of all, we are limited with regard to the overall number of patients to be expected to meet our inclusion and exclusion criteria. Secondly, data for defining a minimally clinically relevant effect is missing and this study may help in providing first phase II evidence to generate such information. Based on a study by the GENERATE registry [18], we observed the following mRS score discharge distribution among 120 patients with severe autoimmune encephalitis after a median hospital stay of 49 days (103 patients with available mRS score-n: 5–17; 4–28; 3–27; 2–19; 1–12). For the non-parametric sample size planning, the effect size is given as relative treatment effect *p* [21]. Under the null hypothesis H0: *p* = 0.5 and under the two-sided alternative H1: *p* ≠ 0.5. For a sample size of 2 × 20 patients, our study has a power of ≥80% to detect strong effects of *p* ≥ 0.75 (or *p* ≤ 0.25) (which roughly corresponds to an effect size of one standard deviation for the case of Students’ *t* test) at a two-sided significance level of 5% for the non-parametric Wilcoxon-Mann-Whitney test as implemented in nQuery Advisor© 7.0. Similar numbers result for the test by Brunner and Munzel which will be used for the primary, confirmatory analysis [21]. We plan to include 2 × 25 patients in case of potential drop-outs.

### Statistical analysis

Two statisticians are involved in data analysis. One statistician is unblinded and provides the randomization lists and any data required by the DSMB. The other statistician is blinded and will perform data analysis. The primary confirmatory analysis will be performed in the ITT analysis set, and the primary endpoint will be compared between both groups by the two-sided Brunner and Munzel test [21]. The significance level *α* of the confirmatory analysis will be set at 0.05. Similar sensitivity analyses are planned for the per-protocol (PP) analysis set. As additional sensitivity analyses, we will try to fit a generalized linear model for the primary endpoint including treatment and autoantibodies group as fixed factors. Multiple imputations of the primary endpoint will be performed to evaluate the sensitivity of the results in case of unexpected missing values. For the comparison of continuous and ordinal secondary endpoints, we plan to apply the two-sided Wilcoxon-Mann-Whitney test. Nominal secondary endpoints will be compared by the two-sided Fisher’s exact test or Freeman-Halton test in case of more than two categories. Since the analyses of the secondary endpoints are all exploratory, the significance level of each test is 0.05, i.e., no correction for multiplicity will be applied. We planned no interim analyses.

### Stopping rules

The study can be terminated for individual patients if the patient or the legal representative withdraws informed consent. The investigator may discontinue a patient’s study participation at any time during the study when the patient meets the study termination criteria:

- Progressive multifocal leucencephalopathy
- Posterior reversible encephalopathy syndrome
- Severe reactions due to immune complexes, e.g., proliferative glomeruolonephritis and polyarthritis
- Grade 4 bortezomib-induced neuropathy (life-threatening) or severe autonomous neuropathy
- Grade 3 bortezomib-induced neuropathy (severe symptoms, activity of daily living significantly affected) or grade 2 neuropathy (moderate symptoms, activity of daily living affected) with pain lasting longer than 2 weeks
- Absolute neutrophil count < 750/μl after pausing bortezomib for 2 weeks
- Absolute thrombocyte count < 25,000/μl after pausing bortezomib for 2 weeks

A study site may be terminated prematurely or suspended if the site and/or the investigator is found in significant violation of GCP, protocol, and contractual agreement, or is unable to ensure adequate recruitment for the study.

The external DSMB will review the progress of the study and perform interim reviews of safety data and provide recommendation to the sponsor whether the nature, frequency, and severity of adverse effects associated with study treatment warrant the early termination or modification of the study in the best interests of the patients. If needed, it would be the sponsor’s decision to terminate the trial.

### Ethical consideration

Approval for the trial was obtained by Germany’s Federal Institute for Drugs and Medical Devices (4043753) and the competent/leading ethics committee of the Jena University Hospital (2019-1523-AMG_ff). Additionally, the local ethics committees of participating sites approved the study protocol by now. Written informed consent will be obtained from all patients according to GCP. For patients in whom consent cannot be obtained because of the underlying disease and/or the use of anesthetic or sedative drugs, informed consent is obtained by the legal representative. If such patient later regains his power of judgment, the patient’s informed consent has to be additionally obtained. If the patient then rejects study participation, all following study procedures are ended.

The trial design takes several patient safety considerations into account. The decision on prescription of the trial drug is at the discretion of the treating physician, and after each cycle of study medication, continuation of treatment has to be reevaluated taking the clinical status and laboratory findings into account. Depending on these evaluations, dose of further treatment has to be adapted or suspended. All trial sites will conduct the study in accordance with German laws and ICH guidelines for Good Clinical Practice (GCP). All study participants are insured according to the requirements of the German Drug Law. The confidentiality of records that could identify the trial subject will be protected, respecting the privacy and confidentiality rules in accordance with the applicable laws. Bortezomib is routinely used in oncology and has also been applied in systemic immunological disorders [13, 22]. Potential side effects are well known. As pretreatment with rituximab is obligatory in the study, patients may have an increased likelihood of infections due to combined B cell and plasma cell depletion. Thus, a close safety monitoring will be performed in the study and patients receive an effective and safe antibiotic and antiviral prophylaxis as recommended. In our clinical experience, consecutive treatment with rituximab and bortezomib did not lead to an increased infection rate. Patients are also screened for bortezomib-induced polyneuropathy. In case of such events, doses of study medication will be reduced or suspended, depending on clinical severity of polyneuropathy (Table 2). In our preliminary experience, polyneuropathy only rarely developed in patients with autoimmune encephalitis upon application of bortezomib, and if so, polyneuropathy was fully reversible [10]. With these safety precautions, a maximum of safety is warranted for the study participants. In addition, potential side effects of the study medication will be addressed and documented with clear safety measures. After completing the trial, no open-label extension phase is planned. Patients remain in expert clinical care at the specialized centers. If further off-label treatment with bortezomib after completion of the study will be performed, this is independent from the study and depends on the clinical evaluation and decision of the treating physician at the respective sites.

## Discussion

Autoimmune encephalitis is a severe and potential life-threatening disorder with risk of residual disability. In patients with severe disease, recovery is long-lasting and includes risk of complications, e.g., due to long-term intensive care therapy [18]. The number of patients correctly diagnosed with these disorders is continuously increasing, but there is no approved medication available and there exists no data from randomized controlled trials. Thus, therapeutic regimen is often guided by expert’s opinion and clinical experience. Treatment of severe cases of autoimmune encephalitis regularly involves treatment with B cell-depleting rituximab. This study will address for the first time in a hypothesis-driven approach whether treatment with bortezomib leads to depletion of plasma cells and reduction of titers of pathogenic antibodies and improves the outcome in patients with severe autoimmune encephalitis. The primary endpoint of the study is the mRS score 17 weeks after first application of the study medication. Although the mRS is not evaluating the multifaceted clinical picture of autoimmune encephalitis, it has become the most widely used clinical outcome measure for stroke clinical trials and has also been used for evaluation in autoimmune encephalitis [18]. Moreover, along the structured evaluation, we will obtain data on disease-related deficits of patients’ memory function and neuropsychological status as well as on the potential beneficial effect of bortezomib on those disease symptoms.

Besides the effect of bortezomib on the functional outcome of the patients, the study will additionally address several other important questions, including the safety of bortezomib in patients with autoimmune encephalitis and pretreatment with rituximab which might be correlated to disease severity and treatment response. With this phase II study, we expect to obtain important information for establishing further hypotheses which may then be investigated by larger phase III trials in collaboration with international clinical networks dedicated to research in autoimmune encephalitis.

### Trial status

The Generate-Boost trial started recruitment in March 2020. The aim is to complete the recruitment by December 2021.

## Supplementary information

**Additional file 1.** SPIRIT 2013 Checklist: Recommended items to address in a clinical trial protocol and related documents.

**Additional file 2:.** Supplementary Table 1. WHO Data Set / Generate-Boost.

**Additional file 3:.** Supplementary Table 2. Primary endpoint. Secondary endpoints.

## Data Availability

The datasets generated and analyzed during the current study are available from the corresponding author on reasonable request. The transfer of data requires a positive vote by the local ethics committee.

## Abbreviations

AEs: Adverse events
BSA: Body surface area
CNS: Central nervous system
CSF: Cerebrospinal fluid
CUS: Sponsor Core Unit
DSMB: Data and Safety Monitoring Board
eCRF: Electronic case report form
GCS: Glasgow coma scale
GFAP: Glial fibrillary acidic protein
GENERATE: German Network for Research on Autoimmune Encephalitis
ICH: International Conference on Harmonization
ICU: Intensive care unit
IVIG: Intravenous immunoglobulin G
ITT: Intention-to-treat
LGI-1: Leucine-rich, glioma inactivated 1
MMSE: Mini-Mental Status Examination
MoCA: Montreal Cognitive Assessment Test
NEOS: Anti-NMDAR Encephalitis One-Year Functional Status
NMDA: *N*-methyl-d-aspartate
NPI: Neuro Psychiatric Inventory
PBMCs: Peripheral blood mononuclear cells
PP: Per-protocol
RAVLT: Rey Auditory Verbal Learning Test
RCT: Randomized, controlled trial
SAE: Serious adverse event
SUSAR: Suspected unexpected serious adverse reaction
SOP: Standard operation procedure
UCH-L1: Ubiquitin carboxy-terminal hydrolase L1
WMW: Wilcoxon-Mann-Whitney
ZKS: Center for Clinical Studies
