# Supplementary material for: Generate-Boost: study protocol for a prospective, multicenter, randomized controlled, double-blinded phase II trial to evaluate efficacy and safety of bortezomib in patients with severe autoimmune encephalitis
Source: Trials. 2020 Jul 8;21:625. doi: 10.1186/s13063-020-04516-7 (PMC7346383; doi:10.1186/s13063-020-04516-7)
Supplement: Supplementary file 2 — Additional file 2:. Supplementary Table 1. WHO Data Set / Generate-Boost. [file 13063_2020_4516_MOESM2_ESM.docx]

**Supplementary Table 1**

**WHO Data Set / Generate-Boost**

| **WHO Data Set Item** | **Answer** | | **Reference** |
| --- | --- | --- | --- |
| **Primary Registry and Trial Identifying Number** | Clinicaltrials.gov  NCT03993262 | | Clinicaltrials.gov registration  Study protocol |
| **Date of Registration in Primary Registry** | 04-SEP-2019 | | Clinicaltrials.gov registration |
| **Secondary Identifying Numbers** | n/a | |  |
| **The Universal Trial Number (UTN)** | n/a | |  |
| **Identifiers assigned by the sponsor** | ZKSJ0120 | | Study protocol |
| **Other trial registration numbers issued by other Registries** | DRKS00017497 | | German Register for Clinical Studies (DRKS) |
| **Identifiers issued by funding bodies, collaborative research groups, regulatory authorities, ethics committees / institutional review boards, etc.** | EudraCT No.: 2019-001423-12 | | EudraCT registration email |
|  | 2019-1523-AMG_ff | | Identifier of leading Ethics Committee |
|  | 4043753 | | Identifier of Regulatory Authority (German Federal Institute for Drugs and Medical Devices – BfArM) |
|  | FKZ 01GM1908E / TP7 | | Identifier of funding body (German Ministry for Education and Research – BMBF) |
| **Source(s) of Monetary or Material Support** | German Ministry for Education and Research – BMBF | | Funding agreement  Study protocol |
| **Primary Sponsor** | Friedrich-Schiller-University Jena (FSU)  With  Prof. Dr. Christian Geis as Sponsor Representative | | Study protocol, agreement over sponsorship and authorization for Prof. Geis to act as sponsor representative on behalf the FSU |
| **Secondary Sponsor(s)** | n/a | |  |
| **Contact for Public Queries** | Prof. Dr. med. Christian Geis  Jena University Hospital, Clinic for Neurology  Section Translational Neuroimmunology  Am Klinikum1, D-07747 Jena  Phone: +49 (0) 3641 9 32 34 13  Telefax: +49 (0) 3641 9 32 34 22  E-Mail: Christian.Geis@med.uni-jena.de | | Study protocol  Registration sites  Website: <https://generate-net.de/boost.html> |
| **Contact for Scientific Queries** | Please see above | | Please see above |
| **Public Title** | n/a – Scientific title is sufficiently understandable for lay public | |  |
| **Scientific Title** | Generate-Boost – A Multicenter Randomized, Controlled, Double-blinded Trial to Evaluate Efficacy and Safety of Bortezomib in Patients With Severe Autoimmune Encephalitis  (Generate-Boost – Multizentrische, randomisierte, kontrollierte und doppelblinde Studie zur Überprüfung der Wirksamkeit und Sicherheit von Bortezomib bei Patienten mit schwerer autoimmuner Enzephalitis) | | Study protocol |
| **Countries of Recruitment** | Germany | | Study protocol |
| **Health Condition(s) or Problem(s) Studied** | Autoimmune encephalitis | | Study protocol |
| **Intervention(s)** | Arms | Assigned Interventions | Study protocol |
|  | Experimental: Interventional | Drug: Bortezomib  1 to 3 cycles Bortezomib with 1,3mg/m^2^ body surface s.c. + 20mg dexamethasone p.o. on days 1, 4, 8 and 11 (= 1 cycle) |  |
|  | Placebo Comparator: Placebo | Drug: Placebo  1 to 3 cycles placebo (NaCl solution) s.c. + 20mg dexamethasone p.o. on days 1, 4, 8 and 11 (= 1 cycle)  Other Names of placebo: isotonic NaCl solution |  |
| **Key Inclusion and Exclusion Criteria** | Inclusion Criteria:   - Age 18 years or older - Clinically diagnosed severe autoimmune encephalitis (mRS ≥ 3) - Antineuronal surface autoantibodies (e.g. NMDA receptor, LGI1, CASPR2, others) in CSF or serum (determined within maximum 4 weeks before randomization) - Pretreatment with rituximab - Written informed consent of the patient or legal representative - Negative pregnancy test in women of child-bearing potential (until 2 years after menopause)   Exclusion criteria:   - Pregnancy/lactation - Acute infiltrating pulmonary disease - Acute infiltrating pericardial disease - Malignant tumor with ongoing chemotherapy - Concomitant participation in other interventional study - Preceding participation in Generate-Boost - Known hypersensitivity to bortezomib or dexamethasone - Ongoing other immunotherapy except for that implicated in the study protocol | | Study protocol |
| **Study Type** | - Prospective - Multicentric - Interventional - Randomized-controlled (1:1 randomization according to computer-based algorithm, stratified according to site. Unblinded biometrician for preparation of randomization lists, blinded biometrician for statistical analysis) - Double-blinded (blinded: treating physician/study team and patient; unblinded: pharmacist. Pharmacist will provide treating physician with ready-to-use syringe with label “for study Generate-Boost only / Bortezomib OR placebo”) - Parallel - Phase II | | Study protocol |
| **Date of First Enrollment** | Enrollment phase started mid of May 2020, but no patient was included until now | | Will be updated in registries as soon as applicable |
| **Sample Size** | Plan to enroll: 50 patients | | Study protocol |
| **Recruitment Status** | Pending: participants are not yet being recruited or enrolled at any site | | Please see point “Date of First Enrolment” |
| **Primary Outcome(s)** | Outcome Name: Modified Rankin Scale (mRS)  Metric/method of Measurement: Scale in points from 0 to 7  Time points: mRS compared from baseline to 17 weeks after first application of study medication) | | Study protocol |
| **Key Secondary Outcomes** | - mRS scores 3, 6, 9 and 13 weeks after first application of study medication / Metric - Glasgow coma scale score (GCS) / 3, 6, 9, 13 and 17 weeks after first application of study medication / metric - Duration of hospital/ duration of ICU stay / over whole study participation time / metric - Neurocognitive function (Montreal Cognitive Assessment Test, MoCA; Mini Mental Status Examination, MMSE; Neuro Psychiatric Inventory, NPI; Rey Auditory Verbal Learning Test, RAVLT) / baseline and 17 weeks after first application of study medication / metric - Absolute and relative change in auto-antibody and vaccine titers in serum and in the cerebrospinal fluid as well as intrathecal synthesis / at baseline and 17 weeks after first application of study medication / metric - Immunostatus (differential blood count, IgG, IgM and IgA fractions) / at baseline and 17 weeks after first application of study medication / metric - Markers of neuronal damage (neurofilament light chain, NFL; Glial fibrillary acidic protein, GFAP; TAU; Ubiquitin carboxy-terminal hydrolase L1; UCH-L1) in the serum / at baseline and 17 weeks after first application of study medication / metric - Change in clonality analysis of B- and T-cell receptors from bulk NGS sequencing from CSF cells and PBMCs / at baseline and 17 weeks after first application of study medication / metric - (Serious) adverse events / from first application up to 17 weeks after first application of study medication / metric - To distinct bortezomib safety: Polyneuropathy, increase of liver enzymes, hemotoxicity, gastrointestinal toxicity, infectious events / from first application up to 17 weeks after first application of study medication / metric | | Study protocol |
| **Ethics Review** | Status: Approved  Date of approval:   - Study protocol Version V01: 29-OCT-2019 - Study protocol Version V02: 13-Jan-2020 - Study protocol Version V03: 04-JUN-2020   Name and contact details of Ethics committee(s):  Ethics Committee of the Jena University Hospital  Bachstraße 18 07740 Jena  Tel.: +49 3641 9-391191 Fax: +49 3641 9-391192 [ethikkommission@med.uni-jena.de](javascript:sendEmail('ethikkommission',%20'med',%20'uni-jena.de',%20'%20')) | | Positive votes /  Study protocol |
| **Completion date:** | Planned completion date: Last Patient, last visit  Anticipated completion date: Q3/2022 | | Study protocol / registries |
| **Summary Results** | n/a | |  |
| **IPD sharing statement** | Decision pending | |  |
