# Supplementary material for: Generate-Boost: study protocol for a prospective, multicenter, randomized controlled, double-blinded phase II trial to evaluate efficacy and safety of bortezomib in patients with severe autoimmune encephalitis
Source: Trials. 2020 Jul 8;21:625. doi: 10.1186/s13063-020-04516-7 (PMC7346383; doi:10.1186/s13063-020-04516-7)
Supplement: Supplementary file 3 — Additional file 3:. Supplementary Table 2. Primary endpoint. Secondary endpoints. [file 13063_2020_4516_MOESM3_ESM.docx]

**Supplementary Table 2**

**Primary endpoint**

| **Domain** | **Specific measurement** | **Specific metric** | **method of aggregation** | **Time point** |
| --- | --- | --- | --- | --- |
| Degree of disability and dependence | mRS score | Change from baseline | Mean/median score | 17 weeks after first application of study medication |

**Secondary endpoints**

| **Domain** | **Specific measurement** | **Specific metric** | **method of aggregation** | **Time point** |
| --- | --- | --- | --- | --- |
| Degree of disability and dependence | mRS score | Change from baseline | Mean/median score | 3, 6, 9 and 13 weeks after first application of study medication |
| Vigilance | Glasgow coma scale score | Change from baseline | Mean/median score | 3, 6, 9, 13 and 17 weeks after first application of study medication |
| Duration of ICU stay | Number of days on ICU | Absolute number | Mean/median score | Discharge from ICU or end of study |
| Duration of Hospital stay | Number of days in Hospital | Absolute number | Mean/median score | Discharge from hospital or end of study |
| Neurocognitive function | Montreal Cognitive Assessment Test, MoCA | Change from baseline | Mean/median score | 17 weeks after first application of study medication |
| Neurocognitive function | Neuro Psychiatric Inventory, NPI | Change from baseline | Mean/median score | 17 weeks after first application of study medication |
| Neurocognitive function | Rey Auditory Verbal Learning Test, RAVLT | Change from baseline | Mean/median score | 17 weeks after first application of study medication |
| Titers  (autoantibody and vaccine titers) in serum | Laboratory results | Change from baseline | Mean/median score | 17 weeks after first application of study medication |
| Titers  (autoantibody and vaccine titers) in CSF | Laboratory results | Change from baseline | Mean/median score | 17 weeks after first application of study medication |
| Immunostatus  (differential blood count, IgG, IgM and IgA fractions) | Laboratory results | Change from baseline | Mean/median score | 17 weeks after first application of study medication |
| Markers of neuronal damage  (NFL; GFAP; TAU; UCH-L1) | Laboratory results | Change from baseline | Mean/median score | 17 weeks after first application of study medication |
| Clonality (B-/T cell receptors) in CSF and PBMCs | Laboratory results (NGS analysis) | Change from baseline | Mean/median score | 17 weeks after first application of study medication |
